# STRFs in primary auditory cortex emerge from masking-based statistics of natural sounds

**DOI:** 10.1371/journal.pcbi.1006595

**Published:** 2019-01-17

**Authors:** Abdul-Saboor Sheikh, Nicol S. Harper, Jakob Drefs, Yosef Singer, Zhenwen Dai, Richard E. Turner, Jörg Lücke

**Affiliations:** 1 Research Center Neurosensory Science, Cluster of Excellence Hearing4all, Department of Medical Physics and Acoustics, University of Oldenburg, Oldenburg, Germany; 2 Zalando Research, Zalando SE, Berlin, Germany; 3 Institute of Biomedical Engineering, Department of Engineering Science, University of Oxford, Oxford, United Kingdom; 4 Department of Physiology, Anatomy and Genetics, University of Oxford, Oxford, United Kingdom; 5 Department of Computer Science, University of Sheffield, Sheffield, United Kingdom; 6 Department of Engineering, University of Cambridge, Cambridge, United Kingdom; 7 Microsoft Research, Cambridge, United Kingdom; University of California at Berkeley, UNITED STATES

## Abstract

We investigate how the neural processing in auditory cortex is shaped by the statistics of natural sounds. Hypothesising that auditory cortex (A1) represents the structural primitives out of which sounds are composed, we employ a statistical model to extract such components. The input to the model are cochleagrams which approximate the non-linear transformations a sound undergoes from the outer ear, through the cochlea to the auditory nerve. Cochleagram components do not superimpose linearly, but rather according to a rule which can be approximated using the max function. This is a consequence of the compression inherent in the cochleagram and the sparsity of natural sounds. Furthermore, cochleagrams do not have negative values. Cochleagrams are therefore not matched well by the assumptions of standard linear approaches such as sparse coding or ICA. We therefore consider a new encoding approach for natural sounds, which combines a model of early auditory processing with maximal causes analysis (MCA), a sparse coding model which captures both the non-linear combination rule and non-negativity of the data. An efficient truncated EM algorithm is used to fit the MCA model to cochleagram data. We characterize the generative fields (GFs) inferred by MCA with respect to *in vivo* neural responses in A1 by applying reverse correlation to estimate spectro-temporal receptive fields (STRFs) implied by the learned GFs. Despite the GFs being non-negative, the STRF estimates are found to contain both positive and negative subfields, where the negative subfields can be attributed to explaining away effects as captured by the applied inference method. A direct comparison with ferret A1 shows many similar forms, and the spectral and temporal modulation tuning of both ferret and model STRFs show similar ranges over the population. In summary, our model represents an alternative to linear approaches for biological auditory encoding while it captures salient data properties and links inhibitory subfields to explaining away effects.

## Author summary

The information carried by natural sounds enters the cortex of mammals in a specific format: the cochleagram. Instead of representing the original pressure waveforms, the inner ear represents how the energy in a sound is distributed across frequency bands and how the energy distribution evolves over time. The generation of cochleagrams is highly non-linear resulting in the dominance of one sound source per time-frequency bin under natural conditions (masking). Auditory cortex is believed to decompose cochleagrams into structural primitives, i.e., reappearing regular spectro-temporal subpatterns that make up cochleagram patterns (similar to edges in images). However, such a decomposition has so far only been modeled without considering masking and non-negativity. Here we apply a novel non-linear sparse coding model that can capture masking non-linearities and non-negativities. When trained on cochleagrams of natural sounds, the model gives rise to an encoding primarily based-on spectro-temporally localized components. If stimulated by a sound, the encoding units compete to explain its contents. The competition is a direct consequence of the statistical sound model, and it results in neural responses being best described by spectro-temporal receptive fields (STRFs) with positive and negative subfields. The emerging STRFs show a higher similarity to experimentally measured STRFs than a model without masking, which provides evidence for cortical encoding being consistent with the masking based sound statistics of cochleagrams. Furthermore, and more generally, our study suggests for the first time that negative subfields of STRFs may be direct evidence for explaining away effects resulting from performing inference in an underlying statistical model.

## Introduction

The goal of this paper is to understand the computational principles which underpin neural processing in auditory cortex. In particular, we investigate the hypothesis that neural processing is shaped by the statistics of natural sounds, the physical rules governing how those sounds combine, and the form of the initial processing performed by the ear.

It is well known that the outer, middle and inner ear transform an incoming sound pressure waveform into a representation at the auditory nerve which can be approximately described by a filtering stage (in which the sound is broken into subbands), followed by an envelope extraction and compression stage. This approximation to the auditory nerve’s representation of a sound is called a cochleagram and intuitively it can be thought of as revealing the spectro-temporal variations in the energy of the input waveform. It is believed that subsequent stages of auditory processing might decompose this representation into basic “structural primitives”, i.e., components or building blocks from which natural sounds are composed. Such a representation would provide a basis to support more complex computation at higher levels in the system (compare, e.g., [1]). The idea of representations in terms of primitives is supported to some extent by *in vivo* recordings in the primary auditory cortex of mammals which suggests that neurons are most sensitive to structures that are localized in time and frequency [2–6], but the hypothesis still lacks convincing evidence.

One way of investigating the hypothesis that auditory cortex is representing the components of natural sounds is to learn their form from a corpus of natural sounds. A particularly popular approach, which has been used for great success for visual data [7] and subsequently for audio data [8, 9], is based on the idea that the stimulus is formed by a linear combination of components which are sparsely activated. However, for auditory stimuli, this “sparse coding” approach is arguably not the most natural one to take for three main reasons. First, a linear mixture of sound pressure waveforms (formed either from multiple sources in the environment or from a single source comprising a linear mixture of primitive components) results in a non-linear mixture at the level of the auditory nerve and it seems likely that downstream processing would respect this fact. Second, the cochleagram is non-negative which is not reflected by the standard form of the sparse coding model. Third, sparse coding (or ICA) operates most effectively on whitened data (although this might be due to current algorithmic limitations, rather than a general feature of the approach).

**Fig 1:**
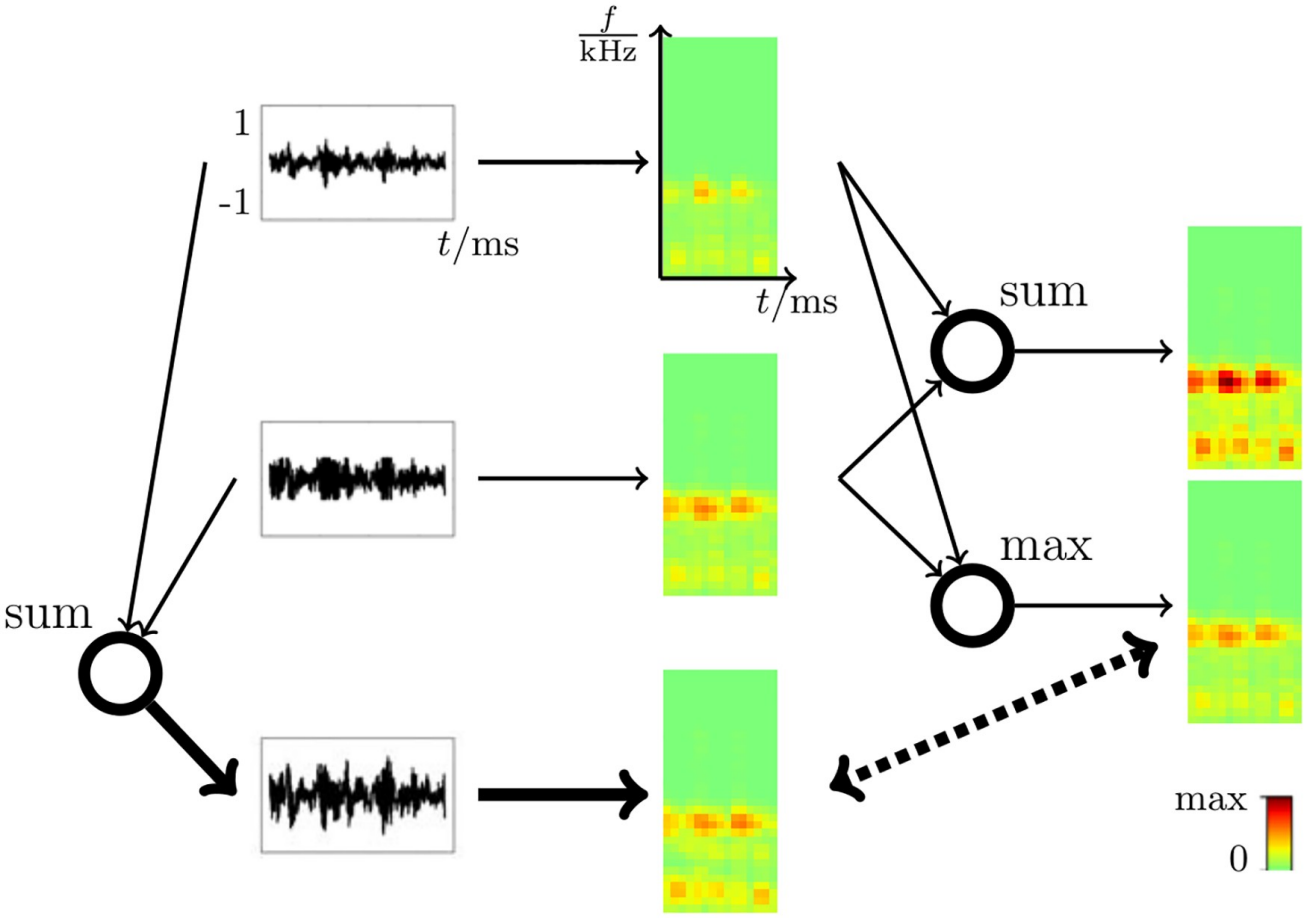
Illustration of the log-max approximation. The figure shows the generation of cochleagrams according to the used preprocessing model and the different combination models (sum and max). First the cochleagrams generated from two different waveforms are shown (middle column, top and middle) as well as the cochleagram generated from the linear mixture of the two waveforms (bottom). On the right at the top, a cochleagram resulting from a linear mixture of the two individual cochleagrams is shown. On the right at the bottom, a cochleagram resulting from a point-wise maximum is shown. The non-linear maximum is much more closely aligned with the cochleagram of the actual mixed waveforms (dotted arrow).

In the visual system it has been argued that the lateral geniculate nucleus (LGN) performs such a whitening step [10] but the initial transformations employed in the auditory system are quite different, making this sort of preprocessing harder to justify. Whitening for cochleagrams would essentially mean that neural activities do not encode energies in frequency bands but deviations from a mean energy relative to energy variances. Adaptation effects to mean and variances over time are well known for regions upstream of the cortex such as the auditory nerve and inferior colliculus [11–14]. However, this adaptation should not be equated with whitening. If it was this would imply that the absence of any signal energy should lead to (on average) equally strong responses as energies above the mean. If we do not assume a whitening stage for cochleagrams or a similar preprocessing to obtain mean-free stimuli, then we are confronted with the question: How do measured STRFs with their positive and negative subfields emerge? In vision, after an assumed whitening stage, stimuli contain positive and negative parts which directly result in components extracted by sparse coding to have negative and positive subfields. For the non-negative energy representation of cochleagrams it is so far unclear how negative subfields can emerge without a whitening stage. Statistical data models not requiring whitening suggest alternative mechanisms commonly referred to as “explaining away effects” which have so far not been linked to negative subfields of neural response properties. As an example for “explaining away” consider the situation of sitting in a park. It is a nice warm day, you have your eyes closed, and are just listening to the sounds around you. There is a small orchestra somewhere with musicians practicing for a concert, and there are birds in the trees. If you now perceive a very short melodic sequence, it may have been generated by a bird or by a musician’s flute. As you are too far away from any of the sources, and as the perceived sequence is too short and unspecific, it is not possible for you to say for sure which of the potential sources may have generated the sound. But you do know that a high probability for one source, e.g. the flute, would mean a low probability for the other. This dependency between the probabilities for the two potential sources given a sound is called “explaining away”. If you were more certain that it was the flute playing (e.g., by getting additional visual input), the flute would “explain away” the alternative explanation of the sound having been generated by a bird. The statistical models investigated here will have similar explaining away effects but on a lower level of sound processing (Fig 6 will give a low level example later on). The primary statistical model investigated here assumes the data to be non-negative (and not whitened), and it assumes the structural primitives to combine non-linearly. More concretely, we assume structural primitives to combine such that the maximal energy in each time-frequency interval determines the superimposed signal (Fig 1 shows an illustration).

To summarize our goal, instead of using the dominating approach of standard sparse coding as statistical model to study neural representation in auditory cortex [8, 9], we investigate for the first time a non-linear and non-negative alternative. Our approach is motivated by the observation that alternatives to the assumptions of linear superposition and whitening may be more natural for acoustic data, and it offers an alternative explanation for the inhibitory subfields of STRFs which were previously closely linked to signal whitening.

## Methods

We will now describe how we change the previously used assumptions of statistical models as discussed above. Engineers have known for a long time that representations such as the cochleagrams result from a non-linear interaction of primitive auditory components. Such non-linear interactions give rise to psychoacoustic masking effects, which have been successfully exploited in technical applications such as source separation (e.g., [15–17]). Underlying such masking effects are that natural sound energies tend to be sparsely distributed across frequencies and time, and that high energies dominate low energies in any spectro-temporal interval of a cochleagram. In practice this property is exploited by assigning each time-frequency interval to the one sound source or component that exhibits maximal energy [15–17], a procedure sometimes referred to as *log-max* approximation. This assumption is widely used in probabilistic models for auditory data processing [15, 16, 18] and finds application in denoising and source separation problems. Here we will also assume a combination rule of this form. Unfortunately, the audio-processing models mentioned above can only handle a small number of components (typically fewer than 10, compare [16]). In contrast, we expect the number of structural primitives required to explain natural sounds to be much larger (similar to a large number of edge-like components required to explain natural images). Therefore, we use instead the relatively novel model of Maximal Causes Analysis (MCA; [19]) that can be scaled to handle hundreds or up to a few thousands of components [20–22]. Not only does this model incorporate the non-linear max combination rule, it also comprises non-negative components much like a non-linear version of non-negative matrix factorization. Importantly, the method performs effectively without need for whitening and so it can be applied directly to non-negative cochleagrams as computed by auditory preprocessing models. The MCA approach, hence, matches those salient features of natural sound statistics previously not captured, making it to a more sensible alternative model for auditory processing in mammals.

### Ethics statement

Animal experiments were done at the Department of Physiology, Anatomy, and Genetics, University of Oxford, performed under license from the United Kingdom Home Office and were approved by the ethical review committee of the University of Oxford. The electrophysiological recordings were made from an adult pigmented ferret under ketamine (5 mg/kg/h) and medetomidine (0.022 mg/kg/h) anesthesia. After recording, the animal was killed with 1ml/kg i.v. Pentoject.

### Models of acoustic preprocessing in mammals

In the inner ear, sound pressure waves are considered to be broken-down into their frequency components by the cochlea, which then also compresses the frequency response amplitudes to form log-spectrograms resembling cochleagram representations of the input signal. The cochleagrams are then further communicated via the auditory nerve for neural processing and as they arrive in higher brain areas such as the primary auditory cortex, the cochleagrams are believed to get decomposed into elementary components for higher-level processing.

#### Cochlear model and spectrogram generation

We model the cochlea of the inner ear as a gammatone filterbank as proposed by Johannesma [23–25]. The time domain impulse function of a gammatone filter is defined as:

$$\begin{array}{c} g\left(t\right)=at^{n-1}\exp\left(-2\pi bt\right)\cos\left(2\pi f_{c}t+\phi \right), \end{array}$$ (1)

where *a* is the amplitude, *b* is the duration of the response, *f*_*c*_ a filter’s center frequency, *ϕ* is the phase and *n* determines the order of the filter. The center frequencies for constructing filterbanks are chosen according to the Equivalent Rectangular Bandwith (ERB) scale, which is proposed by Glasberg [26] based on the physiology of the human ear.

To obtain auditory representations that resemble cochleagrams, we compute the root mean square (RMS) gammatone responses (1) to sound waveforms over a sliding temporal window with an overlapping shift. The RMS energies ${\bar{x}}_{f,t}$ are then passed through a compressive function (i.e., $10\log_{10}\left(1+{\bar{x}}_{f,t}^{2}\right)$) to generate the representations.

### Log-max encoding of cochleagrams

We assume that a cochleagram representation $\vec{y}\in R^{D}$ can be composed as a combination of a (small) number of primitive auditory components ${\vec{W}}_{h}\in R^{D}$, which form elements of a large dictionary $W=({\vec{W}}_{1},\ldots ,{\vec{W}}_{H})$ of *H* components. For such a multi-component encoding scheme, classical modeling approaches such as standard sparse coding [7] or ICA [27, 28] assume a linear interaction of the components to define a data generation process:

$$\begin{array}{c} \vec{y}=\sum_{h}s_{h}\;{\vec{W}}_{h}\;+\;\vec{\eta }\;, \end{array}$$

where $s_{h}\in R$ determines the mixing factors for components ${\vec{W}}_{h}$ and $\vec{\eta }$ denotes added noise in the generative process (which usually is assumed to be zero for ICA). However, cochleagrams are a representation of a non-linear interaction between the auditory components, for which a more accurate generative process can be derived from the log-max approximation [15–17]. The log-max approximation implies that the cochleagram of a linear mixture of sound waves can be well approximated by taking the pointwise maximum of cochleagrams computed from the individual waveforms. Fig 1 illustrates the approximation based on the cochleagram model used in this study. The example shows a better match by the point-wise maximum than by a linear combination. Hence, based on the approximation, we can define the following probabilistic generative model for cochleagrams:

p(s→|Θ)=∏hπsh(1-π)1-shm(Bernoulli) (2)

$$\begin{array}{ccc} p(\vec{y}|\vec{s},\Theta ) & = & N(\vec{y};\;\max_{h}\left\{s_{h}{\vec{W}}_{h}\right\},\sigma^{2}I)\;, \end{array}$$ (3)

where the max operation is applied element-wise, i.e., ${\left({\text{max}}_{h}\left\{{\vec{x}}_{h}\right\}\right)}_{d}={\text{max}}_{h}\left\{x_{dh}\right\}$, and where I denotes the identity matrix. Here we assume the factors *s*_*h*_ ∈ {0, 1} to be Bernoulli distributed, whereas the observed noise is assumed to be Gaussian. Eqs 2 and 3 are a version of the MCA generative model [19, 20]. Parameters of the model are: the frequency *π* with which a component is activated, the variance of the observation noise *σ*^2^, and the generative components or fields ${\vec{W}}_{h}$, which we will later relate to STRFs. For notational convenience Θ = (*π*, *σ*, *W*) denotes the set of all these parameters.

As a control for later numerical experiments with the MCA model, we will also consider a model assuming a standard linear combination of structural primitives. More concretely, we use a model that shares preprocessing, prior, and noise assumption with the MCA model but uses a linear superposition model instead of the point-wise max:

$$\begin{array}{ccc} p(\vec{y}|\vec{s},\Theta ) & = & N(\vec{y};\;\sum_{h}s_{h}{\vec{W}}_{h},\sigma^{2}I)\;. \end{array}$$ (4)

Eq 4 has the standard form of linear sparse coding approaches [7], and is because of the prior (2) a form of Binary Sparse Coding (BSC; [21, 29, 30]).

### Efficient likelihood optimization

Given a set of *N* cochleagrams ${\left\{{\vec{y}}^{\left(n\right)}\right\}}_{n=1,\ldots ,N}$ computed as in Section Cochlear model and spectrogram generation., we now seek parameters Θ* that optimally fit the MCA model to the data. We use likelihood maximization to find the optimal parameters and apply an approximate version of expectation maximization (EM; [31]) for their efficient estimation.

The application of standard maximum a-posteriori (MAP) based approximations is prohibitively suboptimal for the MCA model because the non-linear interaction of components typically results in multi-modal posteriors. An efficient approximate EM approach which can capture multi-modal posterior structure is, however, provided by Expectation Truncation (ET; [20]). ET can be regarded as a variational EM approach, and it has successfully been applied to MCA [21, 22, 32] and many other generative models [33, 34]. ET approximates the computationally intractable full posterior $p(\vec{s}|\vec{y},\Theta )$ by a truncated one [20]:

$$\begin{array}{c} q^{\left(n\right)}\left(\vec{s};\Theta \right)∼p\left(\vec{s}|{\vec{y}}^{\left(n\right)},\Theta \right)\;\delta \left(\vec{s}\in K_{n}\right), \end{array}$$ (5)

where *δ* is an indicator function (i.e., $\delta (\vec{s}\in K_{n})=1$ if $\vec{s}\in K_{n}$ and zero otherwise). If $K_{n}$ is chosen to be small but such that it contains the states with most posterior probability mass, the computation of the expectations in Eq 5 becomes tractable while a high accuracy of the approximations can be maintained [20]. The set $K_{n}$ is, therefore, chosen to consider the subset of the *H*′ most relevant hidden units for a patch ${\vec{y}}^{\left(n\right)}$. Furthermore, at most *γ* of these *H*′ units are assumed to be active simultaneously $|\vec{s}|\leq \gamma$. Please see Efficient Likelihood Optimization in Supporting Information for a formal definition of $K_{n}$.

Parameter update equations for the MCA model have been derived earlier [19, 21, 32]. They are given by:

$$\begin{array}{cc} W_{dh}^{\text{new}}=\frac{\sum_{n}A_{dh}^{\rho }{\left(\vec{s},W\right)}_{q^{\left(n\right)}}y_{d}^{\left(n\right)}}{\sum_{n}A_{dh}^{\rho }{\left(\vec{s},W\right)}_{q^{\left(n\right)}}}, & \begin{array}{cc} A_{dh}^{\rho }\left(\vec{s},W\right)=\left(\frac{\partial }{\partial W_{dh}}{\bar{W}}_{d}^{\rho }\left(\vec{s},W\right)\right), & \left(6\right)\\ {\bar{W}}_{d}^{\rho }\left(\vec{s},W\right)={\left(\sum_{h}{\left(s_{h}W_{dh}\right)}^{\rho }\right)}^{\frac{1}{\rho }}, & \left(7\right) \end{array} \end{array}$$

σnew=1ND∑n⟨∥y→(n)-maxh{shW→h}∥2⟩q(n),iiiiπnew=1HN∑n⟨|s→|⟩q(n), (8)

where the parameter *ρ* in Eq 7 is set to a large value (we used *ρ* = 20) and ‖⋅‖ in Eq 8 denotes the *L*_2_-norm. The learning algorithm for the MCA generative model is thus given by the equations above with expectation values computed w.r.t. the approximate posterior in Eq 5. The linear BSC model, Eqs 2 and 4 is trained analogously to the MCA model with parameter update equations as derived earlier (e.g., [30]). Please see “Efficient Likelihood Optimization” in Supporting Information for more details.

## Results

### Encoding of artificial and natural sounds

We applied our method to male and female anechoic speeches in English, Japanese, Italian, and German. The data also included recordings of natural sounds such as rustling leaves, clattering stones and breaking twigs. More details about the data acquisition procedure are given in Natural Sound Recordings in Supporting Information.

We cut the waveforms of the recordings sampled at 44.1 kHz into snippets of 160 ms with a 32 ms overlap. The snippets were then transformed to cochleagram representations following section “Cochlear Model and Spectrogram Generation”. For the gammatone preprocessing we used a 32−channel filterbank with center frequencies ranging between 1000 and 22050 Hz. In this work we used Slaney’s implementation [35] to apply a 4^*th*^ order gammatone filter. The outputs of the filter were averaged over a 20 ms sliding window with a 10 ms step size. The averaged energies were then compressed through the logarithm (as described earlier) to generate 32 × 15 cochleagrams, that is the energy at 32 center frequencies over 15 consecutive time windows.

We applied the MCA learning algorithm using *H* = 1000 generative fields to a set of *N* = 72800 cochleagrams. Individual cochleagrams were normalized by the *L*_2_-norm of their energies. To find the maximum likelihood parameters Θ approximately, we performed 70 EM iterations of the ET based learning algorithm described in “Efficient Likelihood Optimization”. The truncation parameters *H*′ and *γ* were set to 10 and 6, respectively. We initialized each of the components in the *W* matrix with the mean of the data perturbed by standard Gaussian noise with zero mean and variance set to 1/4th of the variance of the data.

Parameter *σ* was initialized to the square root of the variance of the data and *π* was set to 30/*H* where *H* = 1000. To minimize the possibility of running into local optima, we applied deterministic simulated annealing [36, 37] for the first half of the EM iterations with a linearly decreasing temperature from 10 to 1 (compare [20]). As a control, we also trained the linear BSC model analogously to MCA, i.e., using the same data preprocessing and initialization details as for MCA.

**Fig 2:**
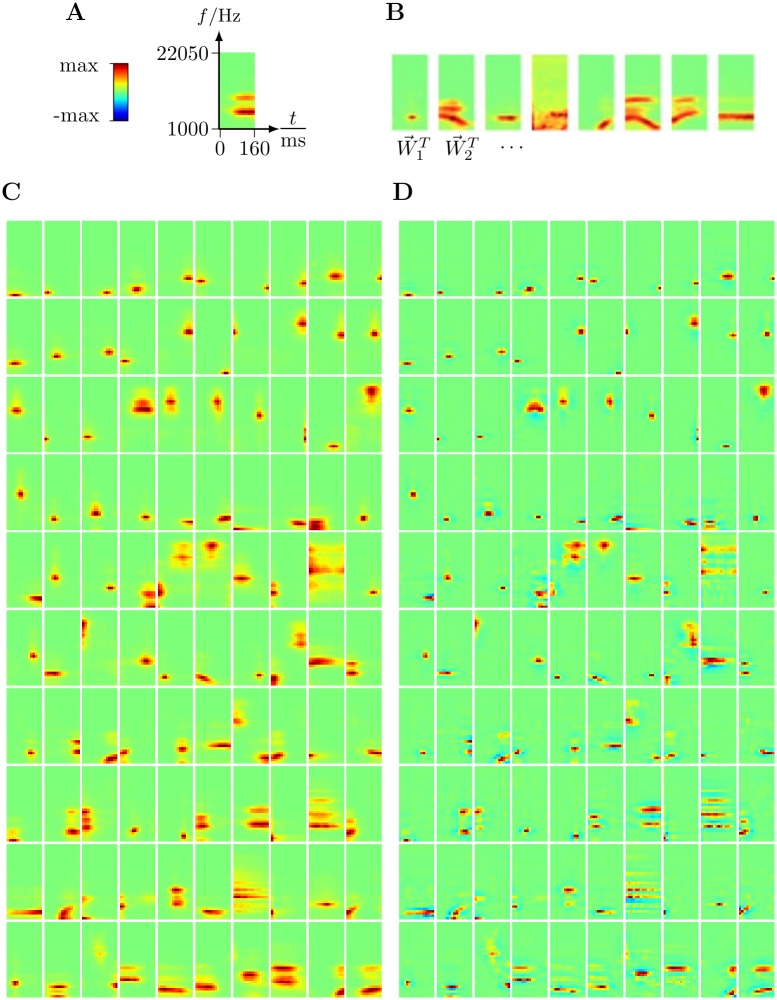
**A-C**: Generative fields learned from the spectrograms of the natural sound data. **A-B**: The vertical axis of the fields are gammatone frequencies with lowest frequency band at the bottom and the horizontal axis spans over 160 ms from left to right. Each generative field is displayed as a 32 × 15 matrix. Fields in panels **A-B** were randomly selected. **C**: Every 5^*th*^ of the 500 most-frequently used fields is shown (ordered w.r.t. their marginal posterior probability from left to right and top to bottom). In total *H* = 1000 fields were learned. **D**: STRF estimates corresponding to the generative fields shown in panel **C**. A larger number of most-frequently employed fields can be found in the supplement, S1 Fig.

Fig 2C shows 100 of the 1000 learned generative fields after the 70 EM iterations. As can be observed, most of the fields are very localized in time and frequency. The generative fields resulting from applying the BSC model are provided in Supplementary S2 Fig.

### Neuronal receptive fields and the encoding in the primary auditory cortex

In order to relate the MCA encoding of cochleagrams to neurons in the auditory cortex, we estimate *spectro-temporal receptive fields* (STRFs) from the inference results of the trained MCA model on the natural sound data. In physiological studies, an STRF is the numerically computed estimation of the linear mapping from sound cochleagrams that best predicts a neuron’s response. Similarly we compute STRFs that we consider to be tuned to individual latent components that we learn. To estimate STRFs ${\hat{W}}^{*}$ for the MCA model, we seek parameters that minimize the following function:

$$\begin{array}{c} f\left(\hat{W}\right)=\frac{1}{N}\sum_{n=1}^{N}\sum_{{\vec{s}}^{\left(n\right)}\in K_{n}}p\left({\vec{s}}^{\left(n\right)}\;|{\vec{y}}^{\left(n\right)},\Theta \right)\;∥\hat{W}{\vec{y}}^{\left(n\right)}-{\vec{s}}^{\left(n\right)}{∥}^{2}+\lambda {∥\hat{W}∥}^{2}, \end{array}$$ (9)

where ${\vec{y}}^{\left(n\right)}$ is the *n*th stimuli, $\hat{W}$ is the row-dominated matrix of predicted STRFs, and λ is the coefficient for L2 regularization. Here we assume that the neural response to a stimulus will be a sample from $p({\vec{s}}^{\left(n\right)}\;|{\vec{y}}^{\left(n\right)},\Theta )$, in which case the experimentally measured STRFs will minimize the squared error between $\hat{W}{\vec{y}}^{\left(n\right)}$ and ${\vec{s}}^{\left(n\right)}$. Our assumption is consistent with interpreting neural responses as posterior samples [38], and the regularization term corresponds to assuming a zero-mean Gaussian hyperprior for the weights (compare ridge regression, e.g., as discussed in [39]). The intractable posterior over the latent factors $p({\vec{s}}^{\left(n\right)}\;|{\vec{y}}^{\left(n\right)},\Theta )$ in Eq 9 is truncated to only cover the subspace $K_{n}$, as defined by the variational approximation technique in Efficient likelihood optimization. By setting the derivative of the cost function (9) to zero, $\hat{W}$ can be estimated as:

$$\begin{array}{c} \hat{W}=(\sum_{n=1}^{N}{\left\langle {\vec{s}}^{\left(n\right)}\right\rangle }_{q_{n}}{\left({\vec{y}}^{\left(n\right)}\right)}^{T}){\left(\lambda NI+\sum_{n=1}^{N}{\vec{y}}^{\left(n\right)}{\left({\vec{y}}^{\left(n\right)}\right)}^{T}\right)}^{-1} \end{array}$$ (10)

where I is the *D* × *D* identity matrix and where ${\langle \cdot \rangle }_{q_{n}}$ denotes the expectation value w.r.t. the approximation $q_{n}\left(\vec{s};\Theta \right)$ of the posterior $p({\vec{s}}^{\left(n\right)}\;|{\vec{y}}^{\left(n\right)},\Theta )$ of the MCA model. The additional term λNI results from a *L*_2_-regularization for *W* in the cost function. Without regularization, the eigenvalues of the data covariance matrix $\sum_{n}{\vec{y}}^{\left(n\right)}{\left({\vec{y}}^{\left(n\right)}\right)}^{T}$ were frequently very close to zero causing numerical instabilities. For the regularization parameter λ, we empirically found that a value in the mid-range of the minimum and the maximum eigenvalues of the data covariance matrix was sufficient to resolve the numerical instability.

Corresponding to the generative fields shown in Fig 2C, Fig 2D illustrates the STRF estimates computed from (10). We will refer to these estimates as *model STRFs* from now on. Observe first that many of the model STRFs are localized in time and frequency, a very common feature of receptive fields in the A1 [3, 40, 41]. Receptive fields produced by earlier sparse coding models do not as extensively have this punctate character [9, 42, 43]. Observe also that many of the model STRFs show flanking inhibition both spectrally and temporally, which is likewise a common feature of A1 receptive fields. However, a difference is that receptive fields of auditory cortical neurons tend to show asymmetry in their temporally flanking inhibition, most inhibition being found in the past relative to the excitatory region.

**Fig 3:**
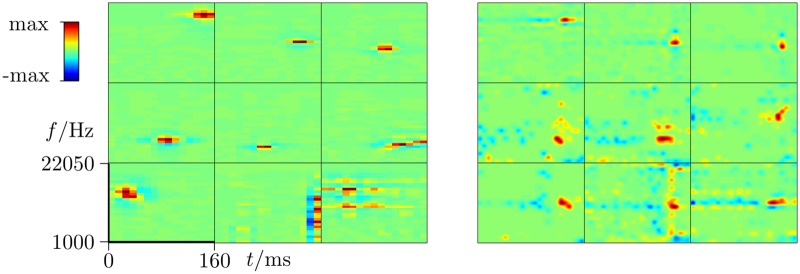
Example receptive fields from the model (left), and similar receptive fields as recorded in ferret A1 (right). The times axis is the x-axis and is from -160 to 0 ms (left) and respectively -125 to 0 ms (right). The frequency axis is the y-axis and is from 1000-22050 Hz (left) and respectively 381-35618 Hz (right), in both cases with lowest frequency at the bottom.

In Fig 3 (left) let us first consider 9 exemplary model STRFs, that illustrate various features which are also seen in experimentally recorded A1 STRFs as illustrated on the right-hand-side of Fig 3 (for how the STRFs were recorded from ferret cortex and estimated see the Supplement). Reading the Fig 3 (left) from left to right, the first unit shows punctate high frequency excitation, the second two units show punctate mid frequency excitation, and the next two units show punctate low frequency excitation. This illustrates that the units’ spectral tuning are spread over the frequency range, as found in physiology, as shown in Fig 3 (right). The sixth unit illustrates an upward sweep in frequency, and the seventh a downward sweep. The eighth and ninth units illustrate receptive fields that are spread out over frequency and time respectively. Again these four types of STRF are found in A1, as show in Fig 3 (right).

**Fig 4:**
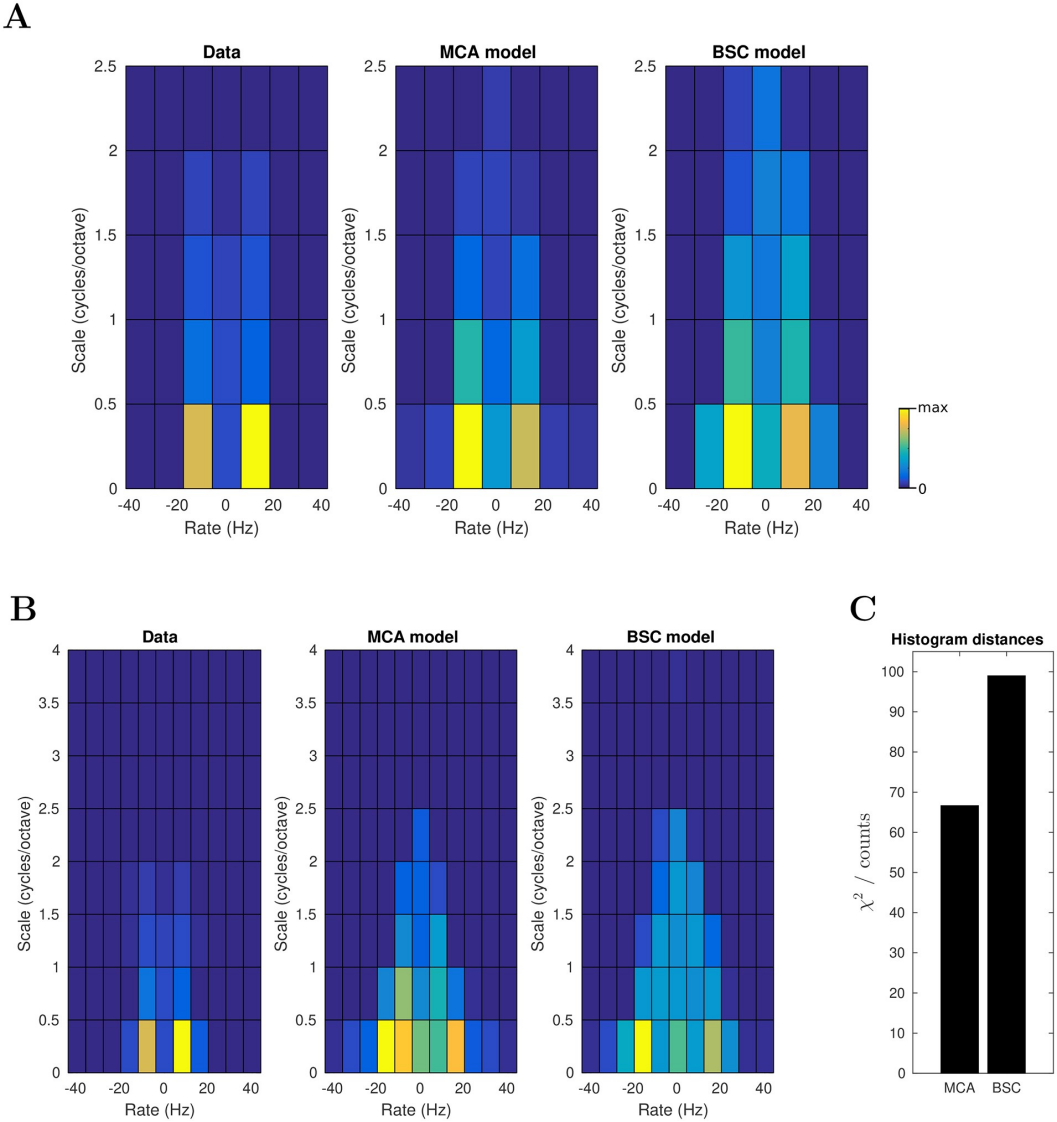
**A**: Histogram of best spectral and temporal modulation frequencies for 241 experimentally recorded STRFs (left) and 241 model receptive fields for the MCA and BSC model (middle and right, respectively). 3/244 recorded STRFs were excluded (see Methods) as they had an L2 norm of zero. Yellow—high density, blue—low density. Histograms are scaled individually to fill the color scale (max is 104 fields for the experimental data, 67 fields for the MCA Model, and 46 fields for the BSC Model). **B**: Histogram shown for a wider range of scales and computed with a bin size of 8 instead of 12 Hz (as used in **A**). Histograms are scaled individually to fill the color scale (max is 78 fields for the experimental data, 36 fields for the MCA Model, and 35 fields for the BSC Model). **C**: For the histograms in **B** a dissimilarity measurement between data and MCA as well as between data and BSC was performed using *χ*^2^ statistics as described in [44].

To quantitatively compare the model STRFs and auditory cortical STRFs across the population we took 244 experimentally recorded STRFs from Ferret A1 and AAF (taken from [45], see Supporting Information: “Neural Recordings and Real STRFs”) and compared them to the most frequently used model STRFs (i.e., to those fields which were the most probable to be activated across all stimuli). For the comparison, a 2D-Fourier transform was applied to each model receptive field and STRF, this provided the modulation transfer function of each receptive field and STRF (3 STRFs were excluded as all their values were zero, see Methods). Then, for each of the 241 remaining real STRFs and model STRFs the frequency modulation and temporal modulation at which the highest value occurred was taken (the best scale and best rate, respectively). A histogram of distribution of best scale and rate is plotted for the real A1 STRFs in Fig 4A and 4B (left), and for the MCA model STRFs in Fig 4A and 4B (middle). The histogram for the BSC model STRFs is shown in Fig 4A and 4B (right). For Fig 4 we used (to match the number of neurons we recorded from) the 241 most frequently used model fields, which represent ≈80% of the overall posterior mass for the MCA model. For comparison, the same histogram but using the 600 most frequently used model fields is shown in the Supplementary S3 Fig (capturing 97% of the posterior mass for the MCA model). S3A Fig (middle) is similar to Fig 4A (middle) but with more model fields at rate zero. The additional fields of Fig S3 which make up the difference to Fig 4 are, however, four times less likely to be active, which makes Fig 4A (middle) more representative for a comparison, see Supplement “Generative Fields and Estimated Model STRFs” for details. In contrast, the histograms for the 241 and the 600 most frequently used BSC fields show comparable percentages of STRFs close to rate zero.

Considering Fig 4, observe that the real STRFs and the receptive fields of the MCA model span a similar range of temporal modulations (rates) and a similar range of spectral modulations (scales). Fields tuned to higher scales and fields with higher and lower magnitudes of rate are a bit more frequent for the MCA model than for the experimental data. For the BSC model, the difference of the histogram to the measured data is larger. Significantly more fields have the best rates around zero or at higher magnitudes than the experimental data. The better match of histogram for the MCA model compared to the linear BSC model can be quantified using a *χ*^2^ test (Fig 4C). In conclusion, the receptive fields of the MCA model and real STRFs span a similar range of temporal modulations (rates) and a similar range of spectral modulations (scales). The model STRFs of the BSC model also span similar ranges of temporal and spectral modulation but this similarity is less pronounced than for the masking-based MCA model.

We also examined the tuning width, over frequency and over time, of the excitatory and inhibitory fields of the real and model STRFs. We used the same most frequently active model fields as for Fig 4, and a tuning width measurement method modified from [46]. For the measurement of frequency tuning width of the excitatory fields, the negative values of the STRFs were set to zero, then the STRF was squared in an element-wise manner and then the STRF was summed over the time bins to give a weighting vector over frequency bands. The excitatory frequency tuning width was then measured as span of frequencies (in octaves) whose weighting was ≥ 50% of the highest weighted frequency channel. For the measurement of temporal tuning width of the excitatory fields, the negative values of the STRFs were set to zero, then the STRF was squared in an element-wise manner, and then the STRF was summed over frequencies, to give a weighting vector over time bins. The excitatory temporal tuning width was measured as the number of time bins that were ≥ 50% of the maximum value of the resulting vector, multiplied by the time bin size of 10 ms. The inhibitory frequency and temporal tuning widths were measured similarly but instead the positive values of the STRF were set to zero, rather than the negative values. For a visualization of how they are measured see Supplementary S4 Fig.

**Fig 5:**
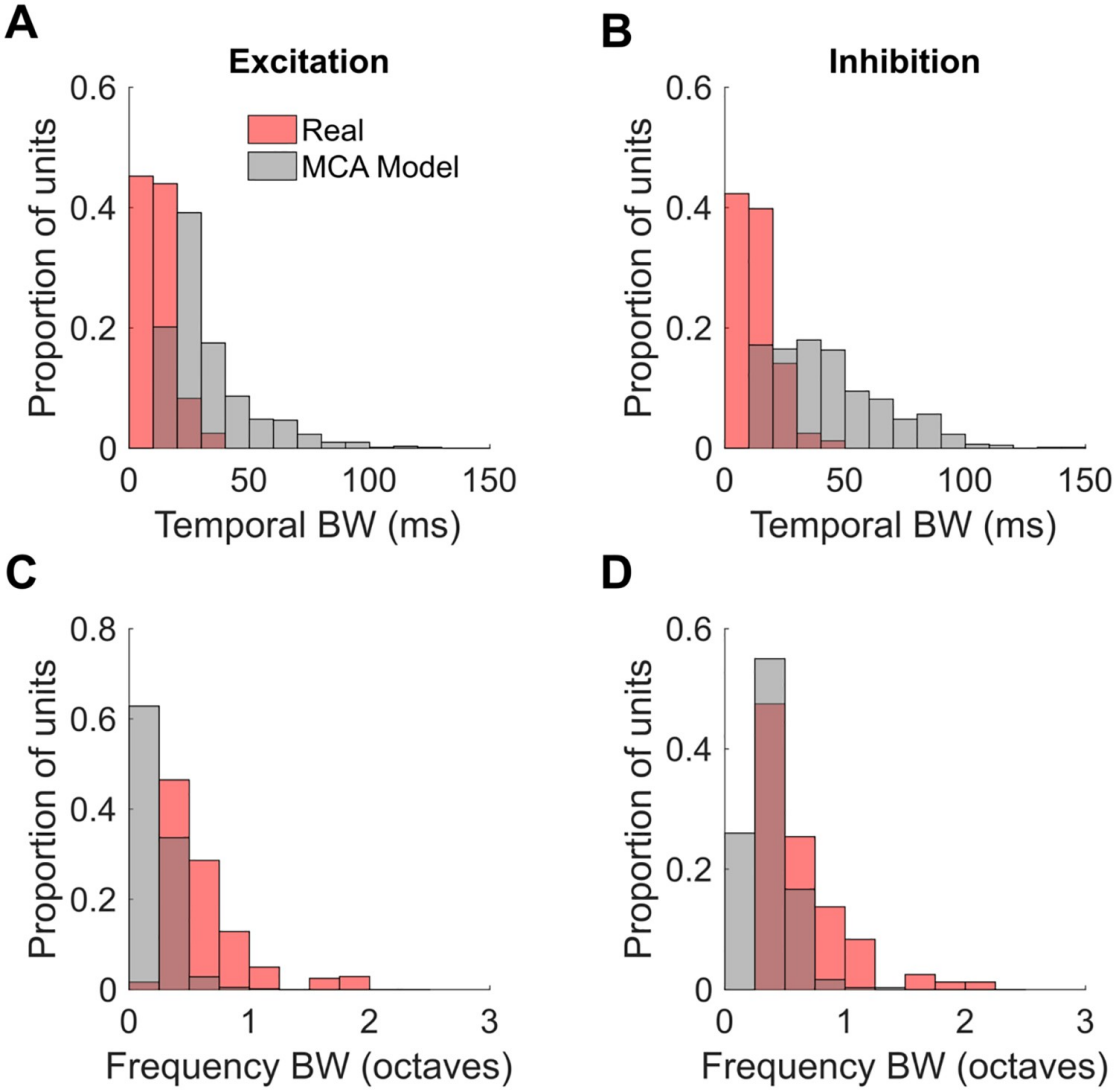
**A**: Distribution over neurons of temporal tuning widths of excitatory fields of the real (pink) and MCA model (grey) neurons. **B**: Distribution of temporal tuning widths of inhibitory fields. **C**: Distribution of frequency tuning widths of excitatory fields. **D**: Distribution of frequency tuning widths of inhibitory fields. For an illustration on how the tuning widths are computed see Supplementary S4 Fig.

Observe that for frequency, for the inhibition and to a lesser extent the excitation, the tuning widths of the MCA model STRFs match relatively well the tuning widths of the STRFs of real neurons. For the temporal dimension we see more strongly diverging properties which may have been expected by considering the statistical modeling approach: Like sparse coding or ICA we do focus on the composition of the data points in terms of structural primitives. Our model itself does not contain statistical dependencies in time unlike hidden Markov models or linear dynamical systems would do. As acoustic data does contain such dependencies on multiple time scales, it is likely that neural processing reflects also these dependencies. The discrepancy of temporal modulation in contrast to frequency modulation may therefore be taken as evidence for the auditory cortex capturing the intricate statistical dependencies over time which neither sparse coding, ICA nor the here studied MCA model addresses. The control experiments using BSC support this interpretation. Also for BSC no asymmetry similar to the one of the measured ferret STRFs is observed. Histograms for BSC computed analogously to Fig 5 are given in the Supplementary S5 Fig. In contrast to the histograms of best modulation frequencies, no notable differences between MCA and BSC histograms were observed.

## Discussion

We have investigated a computational model of auditory processing of sound waveforms in mammals that respects three key constraints. First, that a linear mixture of waveform components results in a non-linear mixing of cochleagram components, which is well approximated by the log-max non-linearity [15, 16]. Second, that the components in the model are positive and sparse. Third, that the statistical model operates on a stimulus closely aligned with biologically processing (cochleagram representation). As such the here followed maximal causes analysis (MCA) approach is arguably a more sensible approach than that provided by linear sparse coding methods that have previously been related to neural STRFs (e.g., [1, 9, 47]), and also of non-negative matrix factorization (NMF; [48, 49]). Perhaps surprisingly, whilst frequently used for sound processing tasks, to the best of our knowledge NMF has not been related to STRF recordings. In fact a relatively recent contribution explicitly states that NMF “does not allow for STRFs with inhibitory subfields” due to the positivity constraint [49].

### Results and predictions

We have shown that the MCA model exhibits a close correspondence to some of the STRF properties of neurons in ferret primary auditory cortex. Like STRFs of the real neurons, the MCA model STRFs show one or a few excitatory regions that are often punctate, being restricted over frequency and often over time. The excitatory regions of the MCA model STRFs are also often flanked by inhibition in frequency and/or time, consistent with real STRFs. The real neurons of our dataset and another ferret cortical dataset [50] show diverse STRFs, likewise the MCA model captures a similar diversity of STRFs with some model STRF broadly tuned over frequency or time, some narrowly tuned, some complex with multiple excitatory regions and some directional with diagonally oriented fields. However, the model STRFs do not capture the fact that inhibitory regions that flank in time tend to occur predominately after excitatory regions, rather than on both sides. This is unsurprising as the MCA model does not have the capacity to reflect causal statistical dependencies in time. MCA shares this property with other ICA-like and sparse coding models (including BSC). It may be noteworthy at this point that already in short-time STRFs, such as we use or are often measured in physiology, the limits of approaches that do not explicitly model dependencies in time are apparent. Measurements and analysis of neural responses in the auditory forebrain of birds [51] suggest that short-time STRFs do represent regularities important for capturing sound regularities over time. There, different types of STRFs have been linked to the processing of different sound properties such as spectral-pitch, rhythm, timbre or periodicity-pitch. Notably, specific functional roles of broad-band STRFs, and of STRFs with inhibition after excitation as well as STRFs with excitation after inhibition have been discussed in this context [51]. Also, the ‘noisy’ type STRFs of Carlin et al [50] with very disordered field structure are not notable in the models here considered.

The control model (BSC) produces STRFs with many properties similar to the MCA model, and most quantitative differences are relatively small. A main difference is that whereas the MCA model reproduces fairly well the distribution of best spectral and temporal modulation frequencies of real neurons, albeit somewhat overestimating the span of rates and scales, the BSC model shows significantly greater overestimation. On other measures they are similar. The MCA model captures fairly well the frequency tuning widths of real neurons, if underestimating to a degree, however in this capacity it did not perform noticeably better than the BSC model. Curiously, although in ferret data and our models the distribution of frequency tuning widths appears unimodal, in bird auditory forebrain [51] the distribution of frequency tuning widths is bimodal, we speculate as a consequence of the statistics of birdsong. Regarding temporal tuning, birds [51], our ferret data, and our models all show apparent unimodal distributions of temporal tuning widths. Both the MCA model and the BSC model substantially overestimate the temporal tuning widths of the STRFs of real neurons, which is again unsurprising as neither model has the capacity to reflect causal statistical dependencies in time.

Furthermore it should be noted that STRFs are far from a complete description of the tuning properties of auditory cortical neurons. Firstly, auditory cortical neurons show many non-linear properties [52] such as conjunctive AND-gate-like behavior [46], or amplitude modulation phase invariance [53]. Secondly, neural tuning properties, including STRFs, can also depend to an extent on stimuli used to gather them [45, 54–59]. Finally, STRFs can also show rapid plasticity depending on the task performed by an awake animal [5].

**Fig 6:**
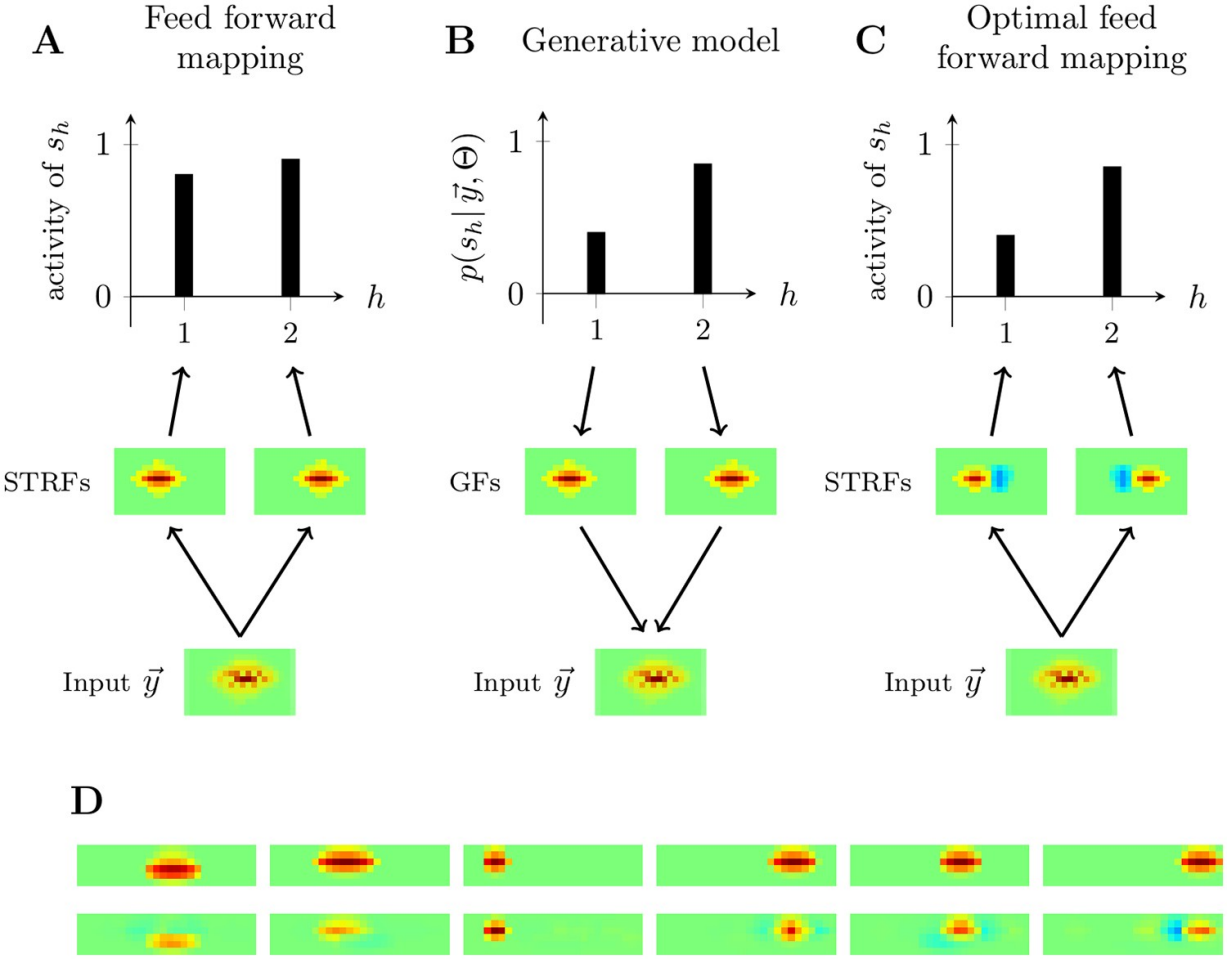
Illustration of the emergence of inhibitory subfields. **A**: Feedforward mapping from an input $\vec{y}$ to two neural units *s*_1_ and *s*_2_. The mapping is defined by two receptive fields with only positive entries. In this case, any strong activation of unit *s*_2_ does not negatively effect unit *s*_1_. For overlapping positive subfields, a stronger activation of *s*_2_ will even result in a stronger activation of *s*_1_ as well. **B**: Activations of neural units *s*_1_ and *s*_2_ according to a statistical model with non-negative generative fields (GFs). Both units compete to explain a presented input $\vec{y}$. A high probability for *s*_2_ decreases the probability of *s*_1_ and vica versa. This effect is known as “explaining away”, and it depends on the assumed model including the model for the combination of primitives, noise model, and prior. **C**: Illustration of an optimal feedforward mapping to approximate neural responses according to the statistical model in **B**. The stronger mutual suppression caused by explaining away is approximated by the introduction of inhibitory subfields. If the input is, e.g., now made stronger or less diffuse, then unit *s*_2_ can increase while unit *s*_1_ can simultaneously decrease, which is in accordance with probabilistic inference for a statistical model. **D**: Example of STRFs estimated from artificial data. The top row shows non-negative GFs. If the corresponding STRFs are now estimated using Eq 10, then negative subfields emerge (bottom row). For fields which do compete little with other fields (e.g., field three) the effect is the weakest. The strongest effects are observed for fields with large overlap (e.g. fields four and six). In general, explaining away effects increase with overcompleteness, i.e., with the number of GFs compared to input size. Color scales for all subfigures as in Fig 2A.

More generally, it is important to acknowledge that comparing normative models such as MCA to real data is difficult and depends on a number of factors including: details of the training corpus, details of different models of preprocessing and details of the STRF estimation. Any of these factors has an influence on quantitative comparisons as those made in this study. For instance, the data used to optimize a statistical model is unlikely to perfectly match the acoustic statistics experienced by the animals used to obtain the experimental data. Or different STRF estimation techniques applied to meet the requirements of experimental recordings or of the used models will effect the quantitative properties of estimated STRFs. Likewise, different preprocessing models (which we have not explored) influence STRF properties (see [60] for a discussion), and have also affected previous work on this topic [8, 9]. Any preprocessing scheme will, however, agree on cochleagrams being representations of acoustic waveform energies in time-frequency intervals. While such representations may be computed by very complex functions, any energy representations will assume non-negative values. Also strong masking non-linearities of the combination of structural primitives within cochleagram representations are widely agreed on in the literature. Notably, although the generative model here considered incorporates the positivity constraint (which we believe is biologically important), the recognition model nevertheless exhibits inhibitory subfields that arise due to explaining away effects among the components. This result indicates, perhaps counter intuitively, that models with positive generative components can still show inhibitory subfields if STRFs for these components’ generative fields are estimated—a finding which has implications beyond the specific model studied here and beyond the auditory system. More precisely, our study shows that inhibitory subfields can be a direct consequence of the statistical model assumed for explaining the data. Even if the data is non-negative and if the used model assumes non-negative generative fields and non-negative latent activities, inhibitory subfields can emerge directly from explaining away effects, without any additional assumptions. Similar to the introductory example, “explaining away” refers to a dependency between alternative explanations for a given stimulus. For our statistical models, possible explanations of a given stimulus take the form of combinations of generative fields (which are typically localized in time and/or frequency). The co-activation of two similar fields is unlikely (because of sparsity) which means that a high probability for one field results in a low probability for the other (and visa versa). Fig 6 aims at providing an intuition why inhibitory subfields emerge because of “explaining away”. Note that the fact that inhibitory subfields do emerge is independent, e.g., of the combination rule assumed by the statistical model, i.e., inhibitory subfields can be obtained for non-linear models of generative field combinations (MCA but also, e.g., noisy-OR models [61]) as well as for linear models. For the linear BSC model, we verified such an emergence of negative subfields also for non-negative weights by running additional experiments. While the BSC model we used for controls showed essentially positive weights, negative entries close to zero of the *W* matrix could be obtained and were obtained in our experiments. To ensure that negative subfields of STRFs also emerge for non-negative weights, we artificially enforced all *W* entries for BSC to be non-negative in our additional numerical experiments. Also in that case STRF estimation by Eq 10 resulted in negative subfields (see Supplement “Efficient Likelihood Optimization” for details).

If measured inhibitory subfields are a consequence of explaining away, then their shapes and the predicted dependencies among hidden neurons change depending on the assumed statistical model. By providing strong evidence for inhibitory subfields to be solely obtainable as a consequence of explaining away, our study offers novel ways of neuro-physiologically evaluating statistical models of neural processing.

Here we have compared spectral and temporal modulation as well as temporal and frequency tuning in order to compare different statistical models with data. Comparison of models is made difficult due to the above discussed factors. Significant differences of predicted STRFs can, nevertheless, be obtained if directly comparing statistical models with and without masking non-linearity (e.g., Fig 4) while all other model properties, training, and preprocessing remained fixed. A step further in the direction of neural evaluation would be represented by a direct *in vivo* comparison of neural responses to specifically designed stimuli. Given a set of neurons with previously measured STRFs, their responses could be predicted based on different statistical models. These different models will predict different response distributions, and artificial stimuli could be designed to be maximally discriminative between any two statistical models. Based on the results of this study, we predict responses for neurons in A1 which compete to explain an acoustic stimulus to *not* show a linear anti-correlation (as predicted by linear models). Explaining away resulting from a masking-based model (such as MCA), in contrast, would predict that neurons explaining the same stimulus compete rather in a k-winner-take-all manner, i.e., small sets of neurons suppress activity in the other neurons with only the maximally active neuron being relevant. For a comparison of explaining away effects between linear models and MCA see e.g. [62], for k-winner-take-all neural circuits see e.g. [63, 64]. In this context, let us, furthermore, remark that any neural activity distribution predicated by a model will not only depend on the model for generative field combinations but also on assumed priors, noise model and on the applied approximate inference approach. Furthermore, it will be important which variables of the model are assumed to match any measured neural activity best. Progress in neural recordings, simultaneous recording and stimulus generation, and refined neural modeling may make a direct comparison of statistical models feasible in the intermediate future.

### Comparison to other normative models

A number of normative approaches have been taken to understand auditory spectro-temporal receptive fields as a consequence of stimulus statistics (e.g. [1, 9, 42, 43, 47, 49, 50, 65, 66]). Before discussing similarities and differences in relation to the models used here, let us stress that the capturing of stimulus statistics is not the only constraint of importance governing the structure of the nervous system. Biophysical constraints such as energy costs or wiring length are also important, as well as other functional constraints such as the role of particular sounds in an animal’s behavior.

Among the stimulus-statistics-based models, the most common approach has been the encoding of spectrogram-like representations of natural sounds subject to a sparsity constraint on the activity of the encoding units. Some sparse normative models balance a constraint for sparsity (or temporal slowness, [50]) while forcing dispersal [43] or decorrelation [42, 50] between the unit responses, and then learn the encoding receptive fields. More relevant for our study are those models which demand sparsity of unit responses while also generatively estimating the spectrograms from the unit activity via learned generative fields [1, 9, 47, 49, 65]. All the above sparsity and slowness models show some capacity to capture certain characteristics of STRFs. We have made explicit comparison of our model to a linear sparse model in the results (Fig 4), as it is the standard leading normative model of sensory coding, and we indicate the particular strengths of our model. The model of Carlin et al. [50] is less directly comparable to our model as it does not involve an explicit generative model. While it does in some ways better explain auditory cortical STRFs than a sparse coding model, it is clear that the MCA model captures certain aspects of the neural data that the slowness model of Carlin et al. does not address. Notably, the Carlin et al. model shows a near uniform distribution of best scales up to 2.5 cycles/octave, this is in contrast to our neural data (and that of Carlin et al.) and the MCA model where the density decays as scale increases (Fig 4).

In general, masking-based non-linearities, i.e., the dominance of one source in any time-frequency bin, is a property of acoustic data that has frequently been used for acoustic data processing (e.g. [16, 67]). In contrast, however, for the task of generatively explaining acoustic data by statistically learned structural primitives, almost all contributions in the literature rely on standard linear models. This applies for studies with functional focus (e.g., NMF-like [68, 69]) as well as for studies explaining neural response properties [1, 9, 49, 65]. The main reason for this strong focus on linear models is presumably related to the challenge of scaling strongly non-linear models to the large sizes required for sensory data. While linear models, e.g. for visual data, are routinely used with hundreds of generative fields / basis functions since about two decades [27, 70–72], non-linear models have been trained at large scales only relatively recently [21, 22, 62]. Earlier non-linear models, e.g., based on a noisy-OR non-linearity [61] or the maximum [19], have not been sufficiently efficient for learning with large numbers of generative fields.

While the approach used here does model masking, we do (as discussed above) not employ a statistical model that captures regularities in time. Other approaches do consider this important aspect of neural processing [66, 73, 74] e.g., to model longer term amplitude modulation structure of acoustic signals [73, 74]. Moreover, incorporating additional temporal statistical regularities is clearly important for acoustic synthesis [75] and might therefore be expected to have a strong effect on the neural representation of sound.

Among the approaches using assumptions formulated in terms of a statistical model, recent work by Yildiz et al. [47] is closely related to the linear models used in our study. That study, like our approach, seeks to explain acoustic stimuli by combinations of structural primitives. The focus by Yildiz et al. is a specific neural circuit implementation for probabilistic inference and learning. The derivation of the neural circuit relies on a *mean field* approximation for efficient inference, an adaptive Markovian dynamics, and a divisive inhibitory interaction among neurons representing structural primitives. The interaction of these mechanisms are shown to result in a stimulus representation with the underlying goal of providing a Bayes optimal explanation using combinations of learned generative fields. While this goal is shared with our approach, the assumed linear combination of primitives is the crucial difference of Yildiz et al. 2016 to our non-linear approach, i.e., they do not model masking. The generative data model underlying Yildiz et al. consequently more closely corresponds to the Binary Sparse Coding (BSC) model which we used as a control (Eqs 2 and 4). However, while Yildiz et al. infer STRFs from the circuit approximation of probabilistic inference, the results of S2 Fig of our study are based on directly inferring model STRFs from the linear BSC model itself. This makes the emergence of inhibitory subfields a direct consequence of the used generative data model, while Yildiz et al. first motivate a divisive form of inhibition to implement approximate probabilistic inference by their suggested circuit. On the other hand, both the here presented study and the study by Yildiz et al., 2016, provide evidence for auditory STRFs emerging from probabilistic inference and learning. Also both studies may be regarded as providing evidence for inhibitory subfields being a consequence of explaining away effects, as first hypothesizes by preliminary results obtained for our study [76]. In terms of concrete neural circuits that may realize such inference and learning, the study by Yildiz et al. 2016 goes very significantly beyond the research questions addressed here. On the other hand, in terms of showing that inhibitory subfields are a direct consequence of probabilistic inference, and in terms of using such fields to discriminate between different statistical models, our study significantly goes beyond the work by Yildiz et al. 2016.

Finally, note further technical but potentially import differences of approximate probabilistic inference applied to our and related approaches. The dominating approach for learning representations in terms of structural primitives are *maximum a posteriori* (MAP) approximations [7], i.e., the stimulus is represented by the latent state (i.e., by the neuron activities) with the highest posterior probability (highest $p(\vec{s}|\vec{y},\Theta )$ in our case). MAP approximations are both scalable and relatively straight-forward to apply, which makes them being very frequently used also for statistical models of acoustic data (e.g., [1, 9]). However, with only maintaining the most probable hidden state for inference, no rich posterior structure is represented: neither correlations, multiple-modes nor any other type of the here very important explaining away effects is captured. In contrast, for our study and for other recent approaches (e.g., [47]) richer posterior representations play an important role. The observation that no previous study using MAP approximations has related inhibitory subfields of STRFs to explaining away effects, indicates that richer posterior representations seem to be required. However, while Yildiz et al. [47] as well as the BSC model used here maintain non-trivial posterior structures, the types of approximations used are different. Yildiz et al. 2016 employ a fully factored variational approximation (i.e., mean field). Such an approximation essentially assumes *a posteriori* independence of neural units, which has (given a stimulus) a direct impact on the activity dependencies among the stimulus encoding neurons. In contrast, the BSC model (as well as the MCA model) uses a truncated EM approximation which does *not* assume *a posteriori* independence [20]. The *a posteriori* independence of mean field has been criticized for introducing biases during learning [77, 78] while approaches that use truncated EM instead have been favorably compared with mean field [34].

### Conclusion

To summarize, we have here shown that statistical models reflecting challenging data properties such as masking-based combinations of structural primitives and non-negativity are applicable to complex sensory data such as cochleagrams. Furthermore, we have found that inhibitory subfields of estimated model STRFs can directly emerge from explaining away effects of the assumed statistical model. This observation may lead to novel tools for the investigation of assumptions underlying probabilistic inference in the auditory cortex, in other sensory areas, and beyond.

## Supporting information

S1 File Details about the Methods and Results sections can be found in this file. (PDF)

S2 File File descriptions and Matlab code can be found in this file. (TXT)

S3 File MCA generative fields can be found in this file. (MAT)

S4 File STRF estimates of MCA can be found in this file. (MAT)

S5 File Generative fields and STRF estimates of the BSC model can be found in this file. (MAT)

S6 File STRF estimates based on measurements in A1 of ferrets can be found in this file. (MAT)

S1 Fig 600 most-frequently used generative and corresponding receptive field estimates obtained with the MCA model. 600 most-frequently used generative and corresponding receptive field estimates obtained with the MCA model. The fields are ordered w.r.t. their marginal posterior probability from left to right and top to bottom.
(EPS)

S2 Fig 600 most-frequently used generative and corresponding receptive field estimates obtained with the BSC model. 600 most-frequently used generative and corresponding receptive field estimates obtained with the BSC model. The fields are ordered w.r.t. their marginal posterior probability from left to right and top to bottom.
(EPS)

S3 Fig Histogram of best spectral and temporal modulation frequencies for experimentally recorded STRFs and BSC model receptive fields. Histogram of best spectral and temporal modulation frequencies for all the 600 model receptive fields shown in S1 Fig (left) and S2 Fig (left), respectively. Model receptive fields were analyzed as in Fig 4 with the same set of measured STRFs for comparison (panel **B** left). Note different y-axis scale in **C**. Color legend as in Fig 4: In **A** max equal to 141 for MCA and 123 for BSC. In **B** max equal to 85 for MCA and 87 for BSC.
(EPS)

S4 Fig Measuring tuning width. Measuring tuning width for Fig 5. **A**: To measure frequency tuning width for the excitatory part of the STRF first an STRF is taken. **B**: Then STRF is element-wise positively rectified and then squared. **C**: Finally the rectified squared STRF is summed over time, and the (not necessarily contiguous) span above half the height is measured to give the frequency tuning width. The frequency tuning width of the inhibitory part is measured the same way, but using negative rectification instead of positive rectification. The temporal tuning width of the excitatory or inhibitory part of the STRF is measured the same way, but with summing over frequency rather than time, and using positive or negative rectification accordingly.
(EPS)

S5 Fig Distribution over experimentally recorded and BSC model neurons of temporal and frequency tuning widths. **A**: Distribution over neurons of temporal tuning widths of excitatory fields of the real (pink) and BSC model (grey) neurons. **B**: Distribution of temporal tuning widths of inhibitory fields. **C**: Distribution of frequency tuning widths of excitatory fields. **D**: Distribution of frequency tuning widths of inhibitory fields.
(EPS)
