# Supplementary material for: STRFs in primary auditory cortex emerge from masking-based statistics of natural sounds
Source: PLoS Comput Biol. 2019 Jan 17;15(1):e1006595. doi: 10.1371/journal.pcbi.1006595 (PMC6382252; doi:10.1371/journal.pcbi.1006595)
Supplement: S1 File — (PDF) [file pcbi.1006595.s001.pdf]

Supporting Information

Efficient Likelihood Optimization

Approximate E-step. The sets $\mathcal{K}_n$ for the approximate posterior (5) are defined along the lines of papers [21, 32]. For completeness, here we briefly describe the definition.

As stated in the main text, a set $\mathcal{K}_n$ is chosen to consider a subset of the H' most relevant hidden units given a patch $\vec{y}$. Additionally, only γ of these H' units are assumed to be active simultaneously $|\vec{s}| \leq \gamma$. Formally, $\mathcal{K}_n$ is therefore defined as follows:

$$\mathcal{K}_n = \{\vec{s} \ \mid (|\vec{s}|_1 \leq \gamma \ \text{ and } \ \forall i \notin I : s_i = 0) \text{ or } |\vec{s}|_1 \leq 1\}\,, \tag{11}$$

where the index set I contains those H' hidden units that are the most likely to have generated data point $\vec{y}^{(n)}$ (while the last term in Eqn. 11 assures that all states $\vec{s}$ with just one non-zero entry are also considered). To determine the index set I, we choose the indices of H' highest scoring hidden units w.r.t. the following *selection function* $\mathcal{S}_h(\vec{y})$:

$$\mathcal{S}_h(\vec{y}) = \mathcal{N}(\vec{y};\, \vec{W}_h^{\text{eff}}, \sigma^2\,\mathrm{I})\,, \quad \text{with an effective weight} \quad W_{dh}^{\text{eff}} = \max\{y_d, W_{dh}\}\,. \tag{12}$$

$\mathcal{S}_h(\vec{y})$ selects hidden components proportional to an upper bound of $p(s_h = 1 \,|\, \vec{y}, \Theta)$ [21, 32]. For the BSC model we can not define a selection based on an upper-bound property (see [20]) but by following previous work [21] we can closely match the selection (12) by using:

$$\mathcal{S}_h(\vec{y}) = \mathcal{N}(\vec{y};\, \vec{W}_h, \sigma^2\,\mathrm{I})\,. \tag{13}$$

Parameters of the approximation are: H' which is the maximal number of components considered per observation $\vec{y}$; and γ which is the maximal number of simultaneously active components γ in any given $\vec{s}$. The values of the parameters are chosen such that the approximate posterior (5) is efficiently computable with a high accuracy. For all experiments on MCA and BSC as reported in the main text, we used $H' = 10$ and $\gamma = 6$.

Finally, in order to show that the emergence of STRFs with negative subfields is not a property exclusive to the non-linear MCA model, we modified the BSC model to be restricted to non-negative fields. In order to do, we clamped after each M-step negative weights W_{dh} to zero. Otherwise, this restricted type of BSC was trained as BSC. Also STRF estimation was done as for unrestricted BSC. The controls verified the emergence of STRFs with negative subfields also for the non-negative BSC model (with STRF properties similar to those of unrestricted BSC, results not shown).

While the approximation used allows the training of large models, the remaining computational costs for training the 1000 GFs for each model, and for estimation of the corresponding STRFs are very extensive. We addressed these costs by executing the learning and estimation algorithms on up to 160 CPU cores on a state-of-the-art compute cluster.

Natural Sound Recordings

The sounds were gathered using a Zoom H2 recorder in an anechoic chamber and also from areas isolated from non-natural sounds in the countryside of Oxfordshire. A highpass 5th-Order Butterworth Filter was applied at 200 Hz to remove any low frequency noise. The gathered files were hand-edited in Audacity to find clean sections with little background noise and clipping.

Altogether there were 131 sound files of variable length. 112 files comprised a total of 1629.3 s of speech in English, Japanese, Italian, and German, spoken by 3 females

and 4 males. 19 files comprised a total of 76.5 s of environmental sounds, consisted of rustling leaves, clattering stones, and breaking twigs. All sounds were recorded in an anechoic chamber.

Neural Recordings and Real STRFs

The neural recordings that were used to provide the STRFs were recorded in response to a stimulus set that was the same as the stimulus set used for the dataset 'comparison' in the study [45]. For more details on the stimulus set and experimental setup we refer the reader to that study. All animal procedures were approved by the local ethical review committee and performed under license from the UK Home Office. In brief, one male adult pigmented ferret underwent electrophysiological recordings under ketamine-medetomidine anesthesia. Extracellular recordings were made from neurons in the primary auditory cortex (A1) and the other primary area, the anterior auditory field (AAF), using silicon probe electrodes (Neuronexus Technologies). These probes had either 32 sites on a single shank, or 8 sites on each of four shanks. The sites were vertically spaced at 50 mm or 150 mm. Stimuli were presented via Panasonic RPHV27 earphones, which were attached to otoscope specula that were inserted into each ear canal, and driven by Tucker-Davis Technologies System III hardware (96kHz sample rate). The stimuli were a diverse range of sounds: two types of dynamic random chords (DRCs), temporally orthogonal ripple combinations (TORCs), modulated noise, and natural sounds. We recorded neural responses to the sounds, presented in random order,and repeated 10 times. The neural spikes were clustered, and those with a sufficient signal to noise power ratio [52] were taken, providing 244 multi-units spike traces, which were used for the STRFs. For each stimulus, peri-stimulus time histograms (PSTHs) were made by counting spikes in 5 ms bins and averaging over repeats. We then characterized the power over frequency and time of each stimulus using a cochlear model (with 21 frequency-channels spanning from 381 Hz to 3.56 kHz). For details of the cochlea model and estimation method of the STRFs (see [45]), which used very similar methods. Using the cochleagrams and PSTHs over all the diverse stimuli, the STRFs were estimated by finding the best linear approximation of the mapping to the PSTHs from the preceding 125 ms of the cochleagrams (The STRFs are the linear part of the linear-nonlinear STRF model of [45]. This was done by minimizing the mean-squared error between the PSTH and its linear estimate from the cochleagram, subject to L1-regularization. The regularization strength was optimized using cross-validation on a randomly-chosen 10% of the data. To perform the STRF fitting we used glmnet for Matlab (J. Quian, T. Hastie, J. Friedman, R. Tibshirani, and N. Simon, Stanford University, Stanford, CA; see `http://web.stanford.edu/~hastie/glmnet_matlab`). For display, STRFs were upsampled by a factor of 4 and then convolved with a 2D 9-point Hann window. To compare the real STRFs with the model STRFs for Fig. 4 (and Fig. S3), as they had different frequency bins and time bins, we convolved the model STRFs with 2D-Gaussians with a standard deviation of 0.5 frequency bins and 0.5 time bins, and after interpolating the data STRFs to have log-spaced frequency bins, we convolved the data STRFs with 2D-Gaussians with a standard deviation of 0.22 frequency bins and 1 time bin, so as to bring their spatial and temporal resolution to a similar level. Also 3 data STRFs were not used in making Fig. 4 (and Fig. S3) as they were zero-valued (L2 norm = 0).

Generative Fields and Estimated Model STRFs

Fig. S1 (left) shows out of the total of 1000 learned fields the 600 most-frequently employed fields for input data encoding used by the MCA model, i.e., the 600 fields

with highest estimated posterior mass $p(s_h = 1 \,|\, \vec{y}^{(n)}, \Theta)$ averaged across all data points. Fig. S1 (right) displays corresponding STRF estimates for the MCA model as computed through regularized reverse correlation (10) in section Neuronal receptive fields and the encoding in the primary auditory cortex. For the BSC model the same is depicted in Fig. S2.

Fig. S3**A-B** (middle) visualizes for the MCA model the spectral and temporal modulation of all 600 model STRFs displayed in Fig. S1 (right). The histogram of the model STRFs is compared to the histogram of the 241 measured STRFs, Fig. S3**A-B** (left), which is the same as Fig. 4**A-B** (left).

Fig. S3**A-B** (right) visualizes for the BSC model the spectral and temporal modulation of all 600 model STRFs displayed in Fig. S2 (right). The histogram of the model STRFs is compared to the histogram of the 241 measured STRFs, Fig. S3**A-B** (left).

Fig. S3**A-B** (middle) is similar to Fig. 4**A-B** (middle) but shows more fields at zero rate. All model STRFs considered for Fig. S3 are treated equally. However, the fields considered by Fig. S3**A-B** (middle) additionally to those of Fig. 4**A-B** (middle) are on average four times less likely to be activated. Rarely active STRFs thus get overemphasized in Fig. S3. The same would be the case even more so if we considered all 1000 fields that were used for the model. The 400 model STRFs that are activated the least only correspond to 2 to 3% of the overall average posterior mass.

Finally, Fig. S4 illustrates how the tuning widths for Fig. 5 are computed. Fig. S5 shows tuning widths for the BSC model.

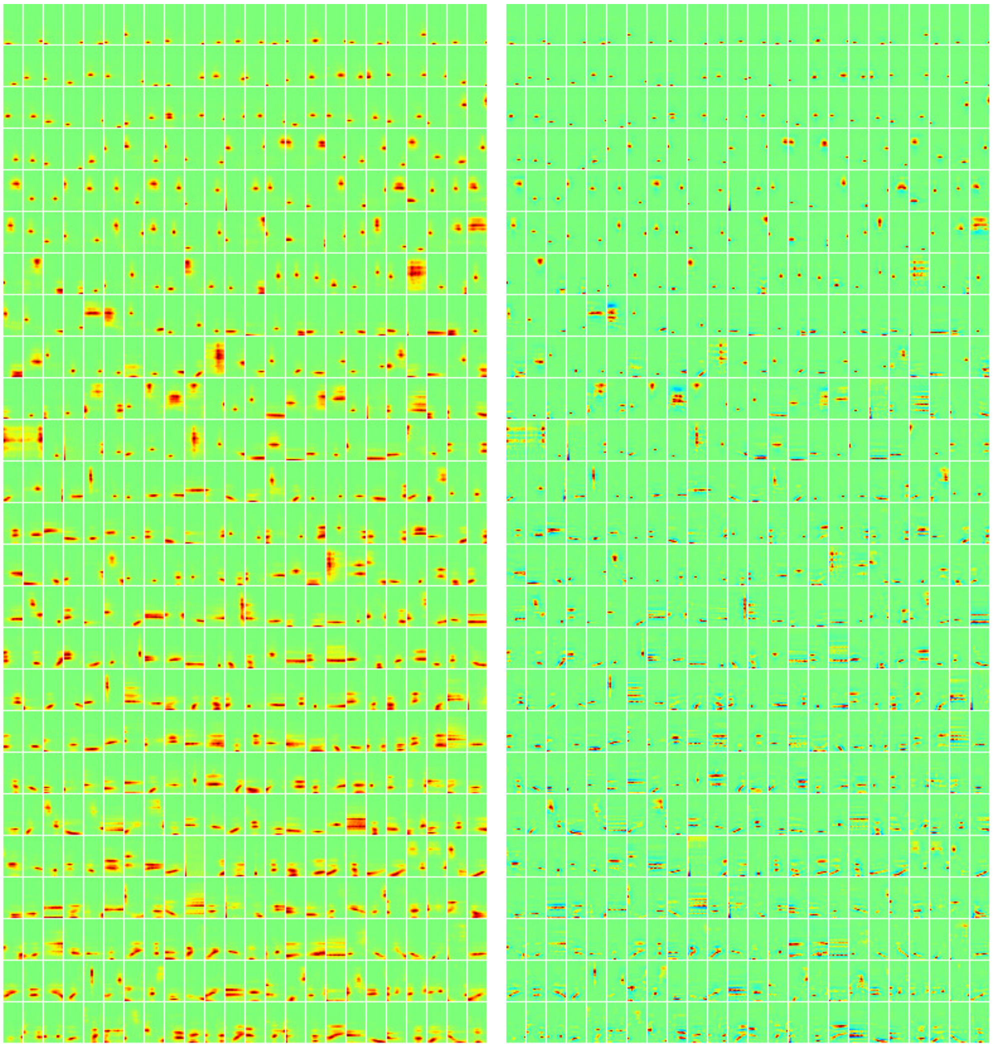

Figure S1. 600 most-frequently used generative and corresponding receptive field estimates obtained with the MCA model. The fields are ordered w.r.t. their marginal posterior probability from left to right and top to bottom.

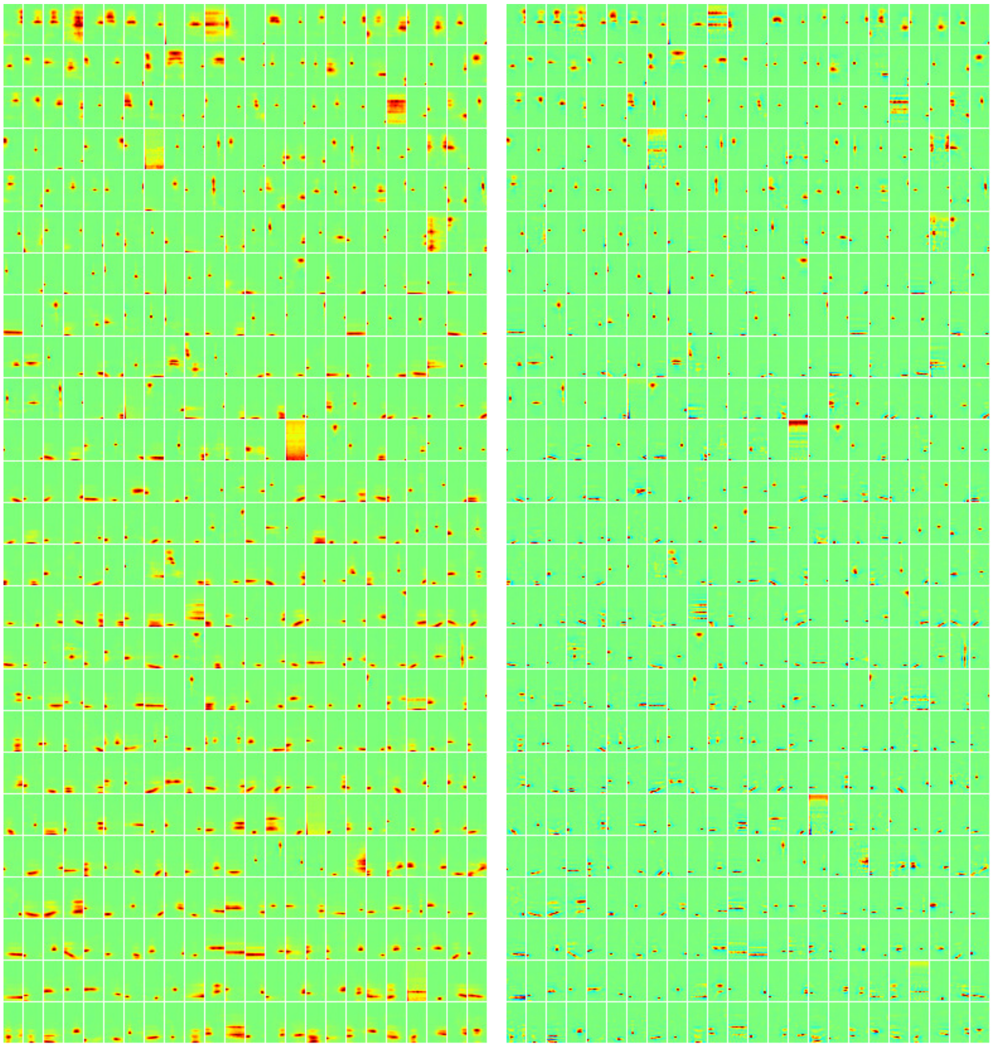

Figure S2. 600 most-frequently used generative and corresponding receptive field estimates obtained with the BSC model. The fields are ordered w.r.t. their marginal posterior probability from left to right and top to bottom.

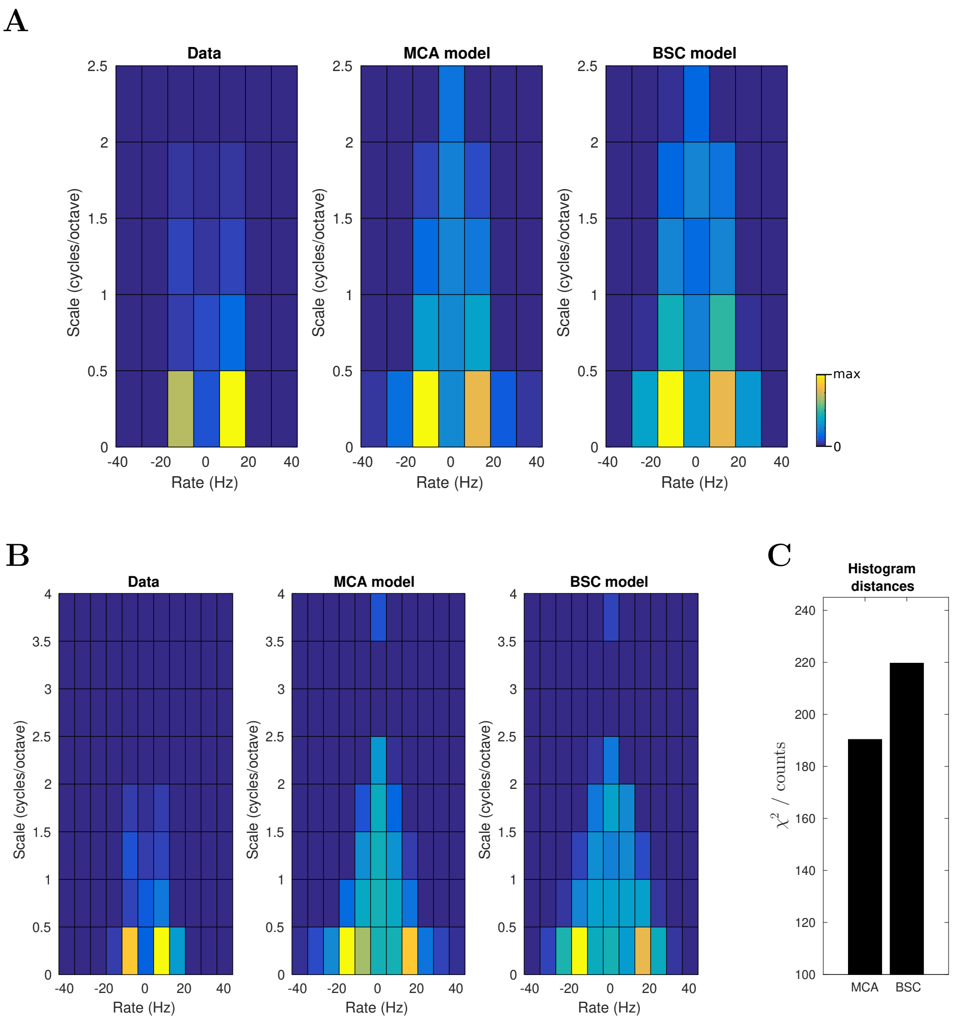

Figure S3. Histogram of best spectral and temporal modulation frequencies for all the 600 model receptive fields shown in Fig. S1 (left) and Fig. S2 (left), respectively. Model receptive fields were analyzed as in Fig. 4 with the same set of measured STRFs for comparison (panel **B** left). Note different y-axis scale in **C**. Color legend as in Fig. 4: In **A** max equal to 141 for MCA and 123 for BSC. In **B** max equal to 85 for MCA and 87 for BSC.

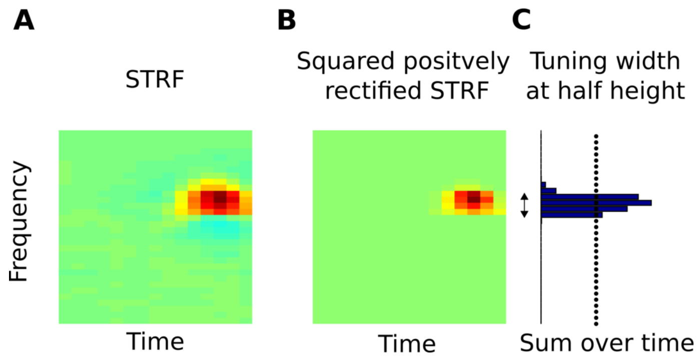

Figure S4. Measuring tuning width for Fig. 5. **A:** To measure frequency tuning width for the excitatory part of the STRF first an STRF is taken. **B:** Then STRF is element-wise positively rectified and then squared. **C:** Finally the rectified squared STRF is summed over time, and the (not necessarily contiguous) span above half the height is measured to give the frequency tuning width. The frequency tuning width of the inhibitory part is measured the same way, but using negative rectification instead of positive rectification. The temporal tuning width of the excitatory or inhibitory part of the STRF is measured the same way, but with summing over frequency rather than time, and using positive or negative rectification accordingly.

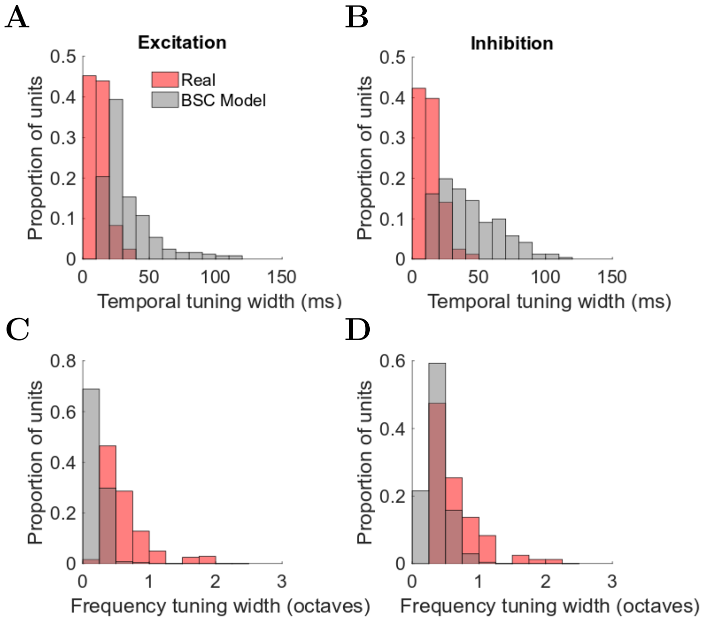

Figure S5. A: Distribution over neurons of temporal tuning widths of excitatory fields of the real (pink) and BSC model (grey) neurons. **B:** Distribution of temporal tuning widths of inhibitory fields. **C:** Distribution of frequency tuning widths of excitatory fields. **D:** Distribution of frequency tuning widths of inhibitory fields.
